# CLINICAL-EPIDEMIOLOGICAL RELATION BETWEEN SARS-COV-2 AND KAWASAKI DISEASE: AN INTEGRATIVE LITERATURE

**DOI:** 10.1590/1984-0462/2021/39/2020217

**Published:** 2020-08-31

**Authors:** Bruna Silva dos Santos, Fernanda Silva dos Santos, Elaine Rossi Ribeiro

**Affiliations:** a Faculdades Pequeno Príncipe, Curitiba, PR, Brazil.; b Universidade Federal de Ciências da Saúde de Porto Alegre, Porto Alegre, RS, Brazil.

**Keywords:** Mucocutaneous lymph node syndrome, Coronavirus infections, Pandemics, Betacoronavirus, Inflammation, Child, Síndrome de linfonodos mucocutâneos, Infecções por coronavírus, Pandemias, Betacoronavírus, Inflamação, Criança

## Abstract

**Objective:**

To analyze the current scientific literature to document, in an integrative review, the main findings that correlate Kawasaki disease (KD) to COVID-19.

**Data sources:**

The search was carried out in June 2020 in the following databases: *Biblioteca Virtual em Saúde* (BVS), *periódico da CAPES* and U.S National Library of Medicine (PubMed). The combination of descriptors used was [(COVID-19 OR SARS-CoV-2) AND (Kawasaki disease)], and the inclusion criteria stipulated were studies published from January 2019 to June 2020, without restriction of language or location, and available online in full. News, editorials, comments, and letters, as well as duplicates and articles that did not answer the guiding question were excluded.

**Data synthesis:**

A total of 97 articles were identified, of which seven comprised this review. The association of KD to the new coronavirus appears to trigger a severe clinical condition of vasculitis. Different from the usual, in this inflammatory syndrome, patients are older, and prevalence is higher in children from African or Caribbean ancestry; clinical and laboratory manifestations are also atypical, with a predominance of abdominal complaints and exaggerated elevation of inflammatory markers. In addition, there was a greater report of rare complications and greater resistance to the recommended treatment for KD.

**Conclusions:**

Pediatric COVID-19 and its potential association to severe KD, still unfamiliar to health professionals, reinforces the importance of testing patients with vasculitis for the new coronavirus and the need to wage high surveillance and preparation of the health system during the current pandemic.

## RESUMO

**Objetivo:**

Analisar a literatura científica atual a fim de documentar, por meio de revisão integrativa, os principais achados que associam a doença de Kawasaki (DK) à doença do coronavírus (COVID-19).

**Fonte de dados:**

A busca ocorreu em junho de 2020, nas bases de dados: Biblioteca Virtual em Saúde (BVS), periódico da Coordenação de Aperfeiçoamento de Pessoal de Nível Superior (CAPES) e U.S. National Library of Medicine (PubMed). Os descritores utilizados foram [(COVID-19 OR SARS-CoV-2) AND (*Kawasaki Disease*)], e os critérios de inclusão estipulados compreenderam estudos publicados de janeiro de 2019 a junho de 2020, sem restrição de idioma ou localização e disponíveis *online* integralmente. Foram excluídos notícias, editoriais, comentários e cartas de apresentação, assim como duplicatas e artigos que não respondiam à pergunta norteadora.

**Síntese dos dados:**

Identificaram-se 97 artigos, dos quais sete compuseram esta revisão. A associação da DK com o novo coronavírus parece desencadear um quadro de vasculite grave. Diferentemente do usual, nessa síndrome inflamatória, os pacientes são mais velhos e a descendência prevalente é africana ou caribenha; as manifestações clínicas e laboratoriais também são atípicas, com predomínio de queixas abdominais e elevação exagerada de marcadores inflamatórios. Além disso, houve maior relato de complicações raras e maior resistência ao tratamento preconizado para DK.

**Conclusões:**

A COVID-19 pediátrica e sua potencial associação com uma DK grave, ainda pouco conhecida pelos profissionais da saúde, reforçam a importância da testagem de pacientes com vasculite para o novo coronavírus e a necessidade de empreender alta vigilância e preparação do sistema de saúde durante a atual pandemia.

## INTRODUCTION

The new severe acute respiratory syndrome coronavirus (SARS-CoV-2) was identified in Wuhan, China, in late 2019, as the cause of COVID-19 and soon became a global health emergency. Most infected adults have a clinical picture with low fever, dry cough, fatigue and odynophagia. Some severe cases progress to acute respiratory distress syndrome, heart failure, hypoxic-ischemic encephalopathy and sepsis.^1^ In contrast, children have milder manifestations and account for only 1-5% of symptomatic cases.^2^ Typical pediatric symptoms include fluctuating fever, signs of upper airway infection, and pneumonia without hypoxemia. Less than 5% of children present severe and critical conditions, characterized by gastrointestinal symptoms, dyspnoea, central cyanosis, acute respiratory failure, and shock.^3^ ^,^ ^4^ ^,^ ^5^ Recently, children infected with SARS-CoV-2 have developed a severe condition of inflammatory syndrome similar to Kawasaki disease (KD), leading to an unusual 30-fold increase in the incidence of this pathology.^6^

KD, first described in 1967 by Tomisaku Kawasaki, is an acute systemic vasculitis that affects small and medium vessels. It predominantly affects children between six months and four years of age (80-90%).^7^ ^,^ ^8^ ^,^ ^9^ Even today, its etiology remains unknown. Hypotheses suggest an infectious trigger in the precipitation of the abnormal immune response associated with genetic susceptibility, as well as a greater racial predisposition in Asians, since the incidence rate in these is 20 times higher than in Caucasians. Although it is a self-limited febrile disease, triggered mainly in winter and of an epidemic character, a pathogen that corroborates the infectious theory has never been identified. New Haven adenovirus, rhinovirus and coronavirus have already been associated to KD, but the results were inconclusive and refuted in later studies.^6^ ^,^ ^7^ ^,^ ^9^

The American Heart Association (AHA) classifies KD in classic and incomplete, or atypical forms. According to the criteria, updated in 2017, the classic form is characterized by the presence of fever lasting five days or more, associated with at least four of the following symptoms:

- Nonexudative conjunctivitis.
- Changes in the lips or oral cavity (erythema, fissures, or flaking).
- Cervical lymphadenopathy.
- Polymorphic rash.
- Changes in extremities in the acute phase (palmar erythema and erythromelalgia), or subacute (periungual desquamation).

Incomplete form is defined as an unexplained fever for five days or more associated with two or three of the classic criteria for classic KD. In infants aged six months or less, the atypical form is determined by unexplained fever lasting seven days or more, even in the absence of classic criteria.^8^ ^,^ ^10^

Potentially fatal complications of KD include macrophage activation syndrome (MAS) and Kawasaki disease shock syndrome (KDSS). MAS is a systemic inflammatory process caused by the activation, proliferation and excessive infiltration of T cells and macrophages, manifesting in only 1.1% of patients.^11^ KDSS refers to a decrease of more than 20% in normal systolic blood pressure, leading to hemodynamic instability. It affects 1.5 to 7% of patients, and should be identified early, because it can progress to shock with strong inflammatory responses that result in coronary artery disease and multiple organ dysfunction.^12^ ^,^ ^13^ Although rare, these complications, when associated with COVID-19, seem to be more prevalent.^14^ ^,^ ^15^

The new coronavirus challenges global health, causing unusual clinical manifestations in previously known and high-incidence diseases. KD demonstrated different characteristics in its typical condition when associated temporarily with SARS-CoV-2. In this context, this review aims to elucidate, based on scientific papers published to date, the relation between SARS-CoV-2 and KD.

## METHOD

This is an integrative literature review, a study that allows the critical evaluation of several methodological approaches, making it possible to gather and synthesize knowledge, as well as draw conclusions based on scientific evidence and apply its results in clinical practice.^16^ ^,^ ^17^

This review aimed to answer the following question: What does the current literature say about the clinical-epidemiological relation between SARS-CoV-2 and KD? After determining the guiding question, the following steps were taken: definition of the database, descriptors and inclusion and exclusion criteria; data collection; evaluation of the titles of selected articles; analysis of the content of abstracts; and careful evaluation and analysis of the articles in full.

The selection of studies was carried out by two independent researchers, in the first week of June 2020, in the following electronic databases: Virtual Health Library (VHL), portal of journals of the Coordination for the Improvement of Higher Education Personnel (*Coordenação de Aperfeiçoamento de Pessoal de Nível Superior* - CAPES), and US National Library of Medicine (PubMed). The descriptors used were previously consulted in the Health Sciences Descriptors (*Descritores em Ciências da Saúde* - DeCS) and Medical Subject Headings (MesH), defining the combination [(COVID-19 OR SARS-CoV-2) AND (Kawasaki Disease)], in search of comprehensive results because of the novelty of the subject. The inclusion criteria defined were studies published from January 2019 to June 2020, without language or localization restrictions, available online in full and with full or partial content approach. News, editorials, comments, and cover letters were excluded.

**Figure 1:**
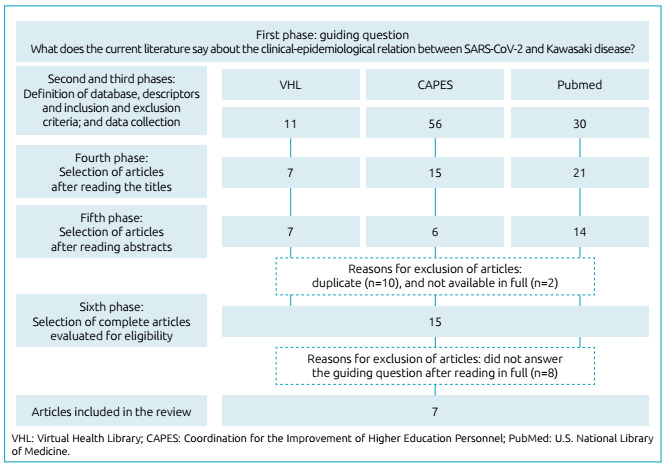
Flow of the selection process of articles for integrative review.

The searches in the VHL (n=11), CAPES (n=56), and PubMed (n=30) resulted in 97 publications. After reading the title, 43 of them were pre-selected for exploratory reading of the abstracts. After a careful analysis, 27 articles remained, of which ten were removed due to duplication, and two because they were not available in full. Thus, 15 articles were selected for complete analysis and, after assessment, seven made up the final sample of this review (Figure 1).

The data of publications were organized and synthesized in the form of a table to simplify the integration of findings, according to the following variables: database, title, journal, author, country/year, and design/sample.

As for ethical aspects, all information extracted from the articles belongs to public domain, and the ideas, concepts and definitions of the authors included in the review were respected.

## RESULTS AND DISCUSSION

As for the country of origin of the selected articles, two are from the United States of America, one from India, and four are from European countries (Italy, France, and England). Regarding the year of publication, all studies were carried out in 2020, and of these, five are from May, and two from June. Regarding the type of study, five are categorized as a case report, and two as a cohort study. All selected articles were written in English. Regarding the areas of knowledge of the journal in which the selected articles were published, four come from pediatric journals (Indian Pediatrics, The Indian Journal of Pediatrics, Hospital Pediatrics, and Journal of the Pediatric Infectious Diseases Society), and three of journals with different themes within the health area (The Lancet and British Medical Journal). Table 1 summarizes these findings.

**Table 1:** General characteristics of the studies included.

Database	Title	Journal	Author	Country/year	Design (n)
PubMed	Kawasaki-like multisystem inflammatory syndrome in children during the COVID-19 pandemic in Paris, France: prospective observational study^18^	British Medical Journal	Toubiana et al.	France, 2020	Prospective observational study (n=21)
VHL PubMed CAPES	An outbreak of severe Kawasaki-like disease at the Italian epicentre of the SARS-CoV-2 epidemic: an observational cohort study^19^	The Lancet	Verdoni et al.	Italy, 2020	Cohort study (n=10)
VHL PubMed CAPES	Hyperinflammatory shock in children during COVID-19 pandemic^20^	The Lancet	Riphagen et al.	England, 2020	Case report (n=8)
VHL PubMed	Multisystem Inflammatory Syndrome in Children during the Covid-19 pandemic: a case series^21^	Journal of the Pediatric Infectious Diseases Society	Chiotos et al.	England, 2020	Case report (n=6)
PubMed CAPES	Incomplete Kawasaki disease in a child with COVID-19^22^	Indian Pediatrics	Rivera-Figueroa et al.	United States, 2020	Case report (n=1)
VHL PubMed	Multisystem Inflammatory Syndrome with features of atypical Kawasaki disease during COVID-19 pandemic^23^	Indian Journal of Pediatrics	Rauf et al.	India, 2020	Case report (n=1)
PubMed	COVID-19 and Kawasaki disease: novel virus and novel case^24^	American Academy of Pediatrics	Jones et al.	United States, 2020	Case report (n=1)

PubMed: U.S. National Library of Medicine; VHL: Virtual Health Library; CAPES: Coordination for the Improvement of Higher Education Personnel.

Data from 48 pediatric patients with KD and suspected SARS-Cov-2 infection were analyzed and compared, 21 of which belong to the study by Toubiana et al.,18 ten by Verdoni et al.,^19^ eight by Riphagen et al., 20 six by Chiotos et al., 21 one by Rivera-Figueroa et al., 22 one by Rauf et al.^23^, and one by Jones et al.^24^ For didactic purposes of the present study, due to the extensive amount of information, eight thematic categories were established: epidemiological characteristics, clinical manifestations, complications, imaging tests, laboratory tests, association between KD and COVID-19, treatment, and pediatric multisystem inflammatory syndrome.

### Epidemiological characteristics

KD predominantly affects children under five years old, average of three years, and boys.^25^ ^,^ ^26^ This review demonstrates that the syndrome, when associated with SARS-CoV-2, seems to affect older children. The studies involved the age range of six months to 16 years, with an average of 7.7 years. As for gender, it was neutral for female and male.

Although this is a disease of worldwide distribution, there is a predominance in the eastern territory and in Japanese descendants living in other continents.^26^ ^,^ ^27^ However, in the current outbreak of COVID-19, there were few cases of inflammatory syndrome in Asian countries, the cradle of the pandemic and KD predominance site.^18^ The ethnic discrepancy between the usual KD and the one associated with the coronavirus was evident here, in which children with African or Caribbean descent represented 43.8% of the cases; Caucasian, 16.6%; Asian, 8.3%; and Middle-eastern, 4.2%. Those with unknown ethnicities accounted for 27.1% of the occurrences.

### Clinical manifestations

KD is characterized by three phases: acute, subacute, and recovery period. Temperature increase and other diagnostic criteria occur in the initial stage.^27^ ^,^ ^28^ Fever for five days or more was found in all patients in this review. The second most prevalent symptom was nonexudative conjunctivitis (83.3%), followed by polymorphic rash (75%), changes in the lips or oral cavity (58.3%), changes in the extremities (56.3%), and cervical lymphadenopathy (29.2%). Specific characteristics of the symptoms were: erythema, fissure, or flaking, present in 100% of those with changes in the lips or oral cavity, whereas strawberry tongue represented 16.7%. Regarding changes in the extremities, 55.6% exhibited erythema or firm hardening, and 60.7% hand or feet edema. According to the 2017 AHA criteria, 62.5% of cases were reported as incomplete or atypical, and 37.5% as complete.

Less common symptoms may be related: abdominal pain (18%), diarrhea (26%), and irritability (50%).^27^ Compared to the review, when associated with SARS-CoV-2, abdominal pain was reported in 89.5% of 38 children,^18^ ^,^ ^20^ ^,^ ^21^ ^,^ ^22^ ^,^ ^23^ ^,^ ^24^ and changes in bowel habits appeared three times more, showing greater gastrointestinal impairment. The presence of meningeal signs associated with KD is little mentioned in the literature. However, research suggests a prevalence of up to 10% of cases of aseptic meningitis.^7^ In this review, neurological changes and signs of meningeal irritation were mentioned in 56.2 and 31.5% of children, respectively.

Variable clinical pictures that appeared in the studies included scrotal edema,^22^ vomiting,^18^ ^,^ ^20^ ^,^ ^21^ arthralgia,^18^ ascites,^20^ tachycardia,^20^ ^,^ ^23^ ^,^ ^24^ tachypnea,^22^ ^,^ ^24^ dyspnea, acute respiratory failure, and respiratory arrest. ^21^

In the subacute phase, nailfold flaking, anorexia, and conjunctivitis can be maintained. In this period, coronary artery aneurysms develop, and the risk of sudden death is greater. Finally, in the convalescence phase, examinations normalize and clinical signs cease, unless complications occur.^7^ ^,^ ^13^ ^,^ ^27^

### Complications

During a complicated clinical picture of COVID-19 and KD, the innate immune system is activated, leading to an exacerbated increase in pro-inflammatory cytokines. Therefore, excessive inflammation and local and systemic damage occur.^13^ ^,^ ^19^ ^,^ ^23^ ^,^ ^29^ The accumulation of inflammatory cells in the endothelial tissue is probably mediated by the angiotensin-converting enzyme 2 (ACE-2), a functional receptor for SARS-CoV- 2. ACE-2 is highly expressed in the alveolar cells of the lung, generating severe pulmonary symptoms. Although the pediatric population has mild respiratory symptoms, the inflammatory response may potentiate the dysfunction of cardiac endothelial cells in cases of pneumonia, leading to coronary lesions and accelerating the development of KD.^29^ ^,^ ^30^

Heart complications, especially myocarditis, occurs early in one third of patients. However, in patients with COVID-19, the damage appears to be greater. Diagnosed by elevating troponin I and reducing the left ventricular ejection fraction, myocarditis occurred in 56.3% of all patients. With myocardial dysfunction and decreased peripheral vascular resistance, some patients may progress with hemodynamic instability and develop KDSS.^18^ ^,^ ^19^ ^,^ ^31^This complication affects from 1.5 to 7% of those with KD.^13^ ^,^ ^18^ Nonetheless, according to the present review, when associated with SARS-CoV-2, its incidence increased, affecting 52.2% out of 38 children.^18^ ^,^ ^19^ ^,^ ^21^ ^,^ ^22^ Hypotension and peripheral hypoperfusion are the main indicators of progression to KDSS, but they are not diagnostic.^12^ ^,^ ^13^ This finding corroborates the fact that 74.1% (n=27) of patients had hypotension, but not necessarily KDSS.^19^ ^,^ ^20^ ^,^ ^21^ ^,^ ^22^ ^,^ ^23^ ^,^ ^24^

MAS has a worldwide incidence of less than 2%.^11^ ^,^ ^33^ Herein, 35.3% out of 17 patients studied had the complication.^19^ ^,^ ^21^ ^,^ ^22^ Although there may be hidden or subclinical MAS, the limited understanding of the disease favors its underdiagnosis in practice.^11^ The clinical hypothesis arises when there is persistent fever associated with splenomegaly, found in 69% of patients,^33^ confronting the fact that only one patient in this review manifested it.^21^

KDSS and MAS were concomitant in three children.^19^ Other independent complications consisted of cardiogenic shock in one patient,^23^ hypovolemic shock in a nine-year-old girl,^21^ and vasoplegic shock in eight patients from a single study.^20^

### Imaging tests

Of all patients who underwent chest radiography (n=47), 19.1% were within normal limits. Reports showed cardiomegaly (8.5%), pneumonia (19.1%), and pulmonary edema (4.3%). In addition, pleural effusion (n=6)^18^ ^,^ ^20^ and radiographic findings compatible with COVID-19 (ground-glass opacity, irregular local shading, and interstitial abnormalities) were found in eight patients.^18^

All patients were submitted to echocardiographic examination. The most seen alteration was a reduced left ventricular ejection fraction in 60.4% of cases, followed by pericardial effusion in 31.3%. The main cardiac complication related to KD is coronary artery aneurysm, which develops in the subacute phase, that is, 14 days after the onset of the disease.^7^ ^,^ ^27^ ^,^ ^30^ In this review, aneurysm (6.3%) and nonspecific abnormalities coronary arteries (22.5%) developed early, according to the average of 8.6 days of hospitalization. Mitral valve dysfunction (12.5%), left ventricular dilation (4.2%), biventricular dysfunction (4.2%), and left ventricular hypokinesia (2.1%) were other findings.

### Laboratory tests

Leukocytosis was observed in 87.5% (n=40) of the cases,^18^ ^.^ ^19^ ^.^ ^21^ ^,^ ^22^ ^,^ ^23^ ^,^ ^24^ lymphocytosis in 78.3% (n=38) of them,^18^ ^,^ ^19^ ^,^ ^21^ ^,^ ^24^ and thrombocytopenia in 63% (n=27) of the total.^19^ ^,^ ^20^ ^,^ ^21^ ^,^ ^21^ ^,^ ^23^ ^,^ ^24^ Anemia was also found in 82.6% of patients (n=23). However, erythrogram was performed in only half of the studies.^18^ ^,^ ^22^ ^,^ ^24^

All investigations tested for inflammatory markers: C-reactive protein (CRP) and erythrocyte sedimentation rate (ESR), which were increased in 97.9% of patients. Besides that, four studies tested for procalcitonin, elevated in 100% out of 35 patients.^18^ ^,^ ^20^ ^,^ ^21^ ^,^ ^22^

Myocarditis is suspected mainly when laboratory levels of troponin I are elevated. In this review, there was an increase in 71.7% out of 46 patients.^18^ ^,^ ^19^ ^,^ ^20^ ^,^ ^21^ ^,^ ^22^ ^,^ ^23^ ^,^ ^31^ Aiming to better consider cardiac function, the plasma dosage of the terminal fragment of the natriuretic peptide type B (n=44, increased by 77.3% ),^18^ ^,^ ^19^ ^,^ ^20^ ^,^ ^21^ the measurement of serum sodium (n=40, reduced by 92.5%)^18^ ^,^ ^19^ ^,^ ^21^ ^,^ ^22^ ^,^ ^23^ ^,^ ^24^, and triglycerides (n=17, increased by 88.2%) were also quantified.^19^ ^,^ ^20^

In order to measure possible lesions in other target organs, creatinine for renal function was assessed, elevated in 44.7% out of 38 patients,^18^ ^,^ ^19^ ^,^ ^21^ ^,^ ^23^ and biomarkers of liver function, such as glutamic-oxalacetic transaminase (GOT) or glutamic-pyruvic transaminase (GPT), were high in 70% out of 40 patients,^18^ ^,^ ^19^ ^,^ ^21^ ^,^ ^22^ ^,^ ^23^ ^,^ ^24^ and plasma albumin reduced in 92.1% out of 38 patients.^18^ ^,^ ^20^ ^,^ ^21^ ^,^ ^22^ ^,^ ^23^

### Association between KD and COVID-19

From 9 to 42% of patients have symptoms of respiratory infection in the 30 days prior to the diagnosis of KD, another piece of information that reinforces the theory of a viral etiopathogenesis. One of the studies in this review precisely illustrates such data, reporting a recent history of flu-like symptoms in an average interval of 45 days between this condition and the signs of KD in 42% of the sample, highlighting the relation between KD and SARS-CoV-2.^18^

Children with SARS-CoV-2 infection should not be compared with those reported in adults, because children seem to have a qualitatively different response to it. Theories explain that the benign course, reduced severity and mortality from the disease in the pediatric population are related to the lower viral load present in the age group, or to the simultaneous presence of other pathogens in the airways, which would limit the growth of the virus.^19^ ^,^ ^29^ In the set of patients in this study, the clinical pictures related to COVID-19 were asymptomatic or with mild respiratory symptoms.^18^ ^,^ ^20^ ^,^ ^21^ Other hypotheses suggest that inflammatory responses of adults and children are discrepant, which is evidenced by the current incidence of multisystemic inflammatory syndrome in children.^29^

A more reliable instrument than the reverse transcription polymerase chain reaction (RT-PCR) for confirmation of active viral infection in patients with KD is the serological test to detect anti-IgG antibodies.^19^ All children in the studies were screened for COVID-19. RT-PCR was positive in 35.4% of them, whereas IgG, investigated in 44 patients, was reactive in 91%.^18^ ^,^ ^19^ ^,^ ^20^ ^,^ ^21^ In the present review, 14 patients presented positive mutual dosages, suggesting a late onset of the syndrome if contrasted with primary infection, and providing data that support the post-viral immune reaction as responsible for KD in predisposed patients.

Both diagnostic tests were negative in four patients;^18^ ^,^ ^19^ another four were tested only for RT-PCR;^21^ ^,^ ^22^ ^,^ ^24^ and one performed the serological test for IgG only after infusion of high doses of immunotherapy, biasing the results.^19^

### Treatment

The standard primary treatment for KD includes acetylsalicylic acid (ASA) and intravenous immunoglobulin (IVIG), starting primarily in the first ten days of fever. The intervention aims not only to control inflammation and subsequent symptoms, but also to prevent long-term cardiovascular sequelae.^7^ ^,^ ^27^ All studies in this review followed the recommended treatment for high-dose IVIG infusion, however ASA was associated in 89,6% of cases.

Considering the importance of early treatment with IVIG to avoid complications, scores were created in an attempt to assume resistance to therapy. Currently, the main one is that of Kobayashi, but this method is not yet precise - approximately 10% remain irresponsible. For AHA, refractory to usual treatment occurs when there is no response to the first infusion, with fever persisting after 36 hours and for less than seven days.^7^ ^,^ ^18^ ^,^ ^27^ ^,^ ^34^ These patients can be treated with a second dose of IVIG or steroids.

As for corticosteroids, it is not yet known whether they are better used as adjuvants or rescue therapy.^7^ ^,^ ^34^ In this review, even with the correct follow-up of the recommended treatment, the rate of resistance and the need for a complementary approach was high, with use of intravenous steroids in 62.5% of patients, proving to be an effective and safe medication for the treatment of KD associated with SARS-CoV-2.

Other drugs used were intravenous antibiotics, in 58.3% of the sample; diuretics for reduction of preload, in 4.2%; and inhibitors of the angiotensin-converting enzyme for reduction of afterload, in 2.1%. Only one article mentioned the use of Anakinra, interleukin-1 receptor antagonist,^21^ and infliximab, a monoclonal antibody, in an eight-year-old child.^20^ Knowing that anaphylactic reaction is one of the adverse effects of IVIG, a patient received intravenous antihistamine as premedication.^22^

Respiratory support was performed in 68.4% of cases (n=38).^18^ ^,^ ^20^ ^,^ ^21^ ^,^ ^22^ ^,^ ^23^ ^,^ ^24^ Hemodynamic support, with inotropes, was reported in 64.6% of all patients. Intensive care unit admission occurred in 32 out of 38 patients.^18^ ^,^ ^20^ ^,^ ^21^ ^,^ ^22^ ^,^ ^23^ ^,^ ^24^

COVID-19 is known for compromising the myocardium, requiring greater cardiac protection for those with cardiovascular comorbidities. For those who manifest myocarditis, echocardiographic monitoring is important at 1-2 and 4-6 weeks after the end of treatment.^24^ ^,^ ^32^ As for other complications, treatment for MAS is usual and, if resistant, tumor necrosis factor inhibitor, immunosuppressive agents or therapeutic plasma exchange must be included. In KDSS, vasoactive drugs have to be associated.^12^ ^,^ ^33^

The average hospital stay calculated between the studies presented was 8.6 days.^18^ ^,^ ^21^ ^,^ ^22^ ^,^ ^23^ ^,^ ^24^ Only one patient remained hospitalized at the end of the study,^21^ and one death from ischemic stroke was reported.^20^

### Pediatric multisystem inflammatory syndrome

The signs and symptoms presented herein, associated with radiographic and laboratory alterations, demonstrate that SARS-CoV-2 infection is generating a severe hyperinflammatory syndrome in pediatric patients, analogous to KD. The probable post-viral pathophysiology of vasculitis and the exacerbation of inflammatory cytokines, present in both COVID-19 and KD, corroborate the hypothesis that the new coronavirus triggers severe KD, presenting marked symptoms and a higher incidence of complications previously described, but that respond to the same therapeutic design.^21^

However, some characteristics are distinguished. Patients with the unusual inflammatory disorder are older and mostly Afro-Caribbean; demonstrate prominent enteropathy and abdominal pain; have overly altered laboratory tests, especially white series and inflammatory markers; show a greater presence of meningeal signs and cardiac involvement; and are more resistant to the usual primary treatment. Thus, despite the similarity with Kawasaki’s severe but rare conditions, and the viable temporality with the coronavirus, combined with the acquisition of immunity, this current manifestation is being provisionally called pediatric multisystemic inflammatory syndrome temporarily associated with SARS-CoV-2 infection., or multisystemic inflammatory syndrome in children.^18^ ^,^ ^21^ Although coronavirus infection in the pediatric population is mild, this condition can be quite rare, but potentially serious.

Children with COVID-19 who have similar clinical characteristics to those of already known inflammatory syndromes, such as KD, are epidemiologically different, clinically more severe and more resistant to treatment. The present review demonstrates that SARS-CoV-2 builds an anomalous immunological reaction, particularly strong, when compared to other infectious agents, constituting a rare and complicated KD due to the associated pathological mechanisms. Nonetheless, studies on the multiplicity of manifestations of KD and research to describe and characterize the COVID-19 infection process in pediatric patients are still needed.

Outbreaks of this hyperinflamatory syndrome, still partially known, can occur in all countries affected by the pandemic, being outside the standard KD phenotype and causing important public health implications. Especially in Brazil, where there is a large Afro-descendant population, the disease must be seriously considered, because it points to the need to implement child reintegration policies. Health professionals need to be aware of these atypical presentations, and hospitals, structurally prepared. The condition needs fast and aggressive management. Otherwise, if not well managed, it can be serious and lethal.

Finally, with the possibility of severe KD manifesting after an immune response acquired by SARS-CoV-2, the concept of a benign COVID-19 in children is not justified. The currently-called pediatric multisystemic inflammatory syndrome temporarily associated with SARS-CoV-2 infection, or multisystemic inflammatory syndrome in children, has much to be explored by clinicians and researchers.
